# Sudden cardiac death with normal coronaries: cardiac MRI in the differential diagnosis of underlying disease in survivors

**DOI:** 10.1186/1532-429X-11-S1-O14

**Published:** 2009-01-28

**Authors:** Peter Hunold, Thomas Schlosser, Kai Nassenstein, Oliver Bruder, Holger Eggebrecht, Peter W Radke, Jörg Barkhausen

**Affiliations:** 1 University Hospital Schleswig-Holstein, Campus Lübeck, Lübeck, Germany; 2 University Hospital Essen, University of Duisburg-Essen, Essen, Germany; 3 Elisabeth Hospital, Essen, Germany; 4 West German Heart Center, University of Duisburg-Essen, Essen, Germany

**Keywords:** Cardiomyopathy, Sarcoidosis, Sudden Cardiac Death, Late Gadolinium Enhancement, Hypertrophic Cardiomyopathy

## Introduction

Sudden cardiac death (SCD) is most commonly caused by acute myocardial infarction as a correlate of coronary artery disease. Therefore, survivors of SCD undergo cardiac catheter to treat or rule out CAD. However, in cases with normal coronary arteries SCD often remains unexplained. Diagnostic work-up in this collective is important to adjust and optimize therapy.

## Purpose

Aim of this study was to evaluate the use of contrast-enhanced cardiac MRI (CMR) in defining the underlying pathology of survived SCD in patients without coronary artery occlusion.

## Methods

More than 6.000 contrast-enhanced CMR studies from 3 different hospitals were reviewed for cases of survived SCD with angiographic rule out of obstructive coronary artery disease. The CMR protocol (1.5 T) consisted of a functional left ventricular study using a segmented SSFP sequence (TrueFISP, balancedFFE) in long and short axes. Data sets for late gadolinium enhancement detection were acquired 8–15 min after 0.2 mmol/kg BW of Gd-based contrast material using a segmented inversion-recovery TurboFLASH/FGRE sequence (TI, 200–260 ms; slice thickness, 8 mm, 2D or 3D). All cases of non-coronary SCD were reviewed and the different underlying pathologies as defined by MRI were collected.

## Results

In total, 18 cases of unclear SCD were identified. In 14 patients thereof, MRI could state the diagnosis based on the typical imaging features of myocardial disease: Primary cardiomyopathy was found in 7 patients (arrhythmogenic right ventricular cardiomyopathy, 2; dilated cardiomyopathy, 3; hypertrophic cardiomyopathy, 1; isolated left ventricular non-compaction, 1). Acute myocarditis and acute cardiac sarcoidosis were found in 3 patients each. Chronic aneurysm of the anterior wall most probably due to cardiac contusion was found in 1 patient. In 4 patients, CMR could clarify the etiology of SCD.

## Conclusion

Contrast-enhanced CMR has unique features in detecting and differentiating myocardial disease with possibly fatal outcome. It has proven to be an utmost valuable tool for the diagnostic work-up of survivors of unclear SCD. This underlines the role of CMR as the first-line technique in myocardial disease.

